# Human bronchial carcinoid tumor initiating cells are targeted by the combination of acetazolamide and sulforaphane

**DOI:** 10.1186/s12885-019-6018-1

**Published:** 2019-08-30

**Authors:** Reza Bayat Mokhtari, Narges Baluch, Evgeniya Morgatskaya, Sushil Kumar, Angelo Sparaneo, Lucia Anna Muscarella, Sheyun Zhao, Hai-Ling Cheng, Bikul Das, Herman Yeger

**Affiliations:** 1 Developmental and Stem Cell Biology Program, The Hospital for Sick Children, Toronto, ON Canada; 2 Department of Paediatric Laboratory Medicine, The Hospital for Sick Children, Toronto, ON Canada; 3 Institute of Medical Science, University of Toronto, Toronto, ON Canada; 4 Department of Pediatrics, Queen’s University, 76 Stuart St, Kingston, ON K7L 2V7 Canada; 5 Department of Pharmaceutical Sciences, College of Pharmacy, University of Nebraska Medical Center, Williams Science Hall 3035, Department of Pharmaceutical Sciences 601 S. Saddle Creek Rd, Omaha, NE 68106 USA; 6 Laboratory of Oncology, Fondazione IRCCS Casa Sollievo della Sofferenza, viale Cappuccini, 71013 San Giovanni Rotondo, FG Italy; 7 Institute of Biomaterials & Biomedical Engineering, University of Toronto, 164 College Street, Rosebrugh Building, Room 407, Toronto, ON M5S 3G9 Canada; 8 Thoreau Laboratory for Global Health, M2D2, University of Massachusetts-Lowell, Innovation Hub, 110 Canal St, Lowell, MA 01852 USA; 9 KaviKrishna Laboratory, Indian Institute of Technology Complex, Guwahati, India; 10 The Hospital for Sick Children, Peter Gilgan Centre for Research and Learning, 686 Bay St., Rm 15.9714, Toronto, Ontario M5G 0A4 Canada

**Keywords:** Bronchial carcinoid, Acetazolamide, Sulforaphane, Orthotopic lung model, Combination therapy, 3D spheroids

## Abstract

**Background:**

Bronchial carcinoids are neuroendocrine tumors that present as typical (TC) and atypical (AC) variants, the latter being more aggressive, invasive and metastatic. Studies of tumor initiating cell (TIC) biology in bronchial carcinoids has been hindered by the lack of appropriate in-vitro and xenograft models representing the bronchial carcinoid phenotype and behavior.

**Methods:**

Bronchial carcinoid cell lines (H727, TC and H720, AC) were cultured in serum-free growth factor supplemented medium to form 3D spheroids and serially passaged up to the 3rd generation permitting expansion of the TIC population as verified by expression of stemness markers, clonogenicity in-vitro and tumorigenicity in both subcutaneous and orthotopic (lung) models. Acetazolamide (AZ), sulforaphane (SFN) and the AZ + SFN combination were evaluated for targeting TIC in bronchial carcinoids.

**Results:**

Data demonstrate that bronchial carcinoid cell line 3rd generation spheroid cells show increased drug resistance, clonogenicity, and tumorigenic potential compared with the parental cells, suggesting selection and expansion of a TIC fraction. Gene expression and immunolabeling studies demonstrated that the TIC expressed stemness factors Oct-4, Sox-2 and Nanog. In a lung orthotopic model bronchial carcinoid, cell line derived spheroids, and patient tumor derived 3rd generation spheroids when supported by a stroma, showed robust tumor formation. SFN and especially the AZ + SFN combination were effective in inhibiting tumor cell growth, spheroid formation and in reducing tumor formation in immunocompromised mice.

**Conclusions:**

Human bronchial carcinoid tumor cells serially passaged as spheroids contain a higher fraction of TIC exhibiting a stemness phenotype. This TIC population can be effectively targeted by the combination of AZ + SFN. Our work portends clinical relevance and supports the therapeutic use of the novel AZ+ SFN combination that may target the TIC population of bronchial carcinoids.

## Background

Bronchial carcinoids are a more indolent subgroup of neuroendocrine tumors (NETs) that arise in the lateral region of the bronchus. The slower growth of bronchial carcinoids generally portends a better prognosis but is dependent on the degree of differentiation. Bronchial carcinoids present as typical carcinoids, TC, or a more aggressive form, atypical carcinoids, AT. TC tumors are well-differentiated, rarely metastasize, and have a good prognosis with a survival rate of 87 to 100% [1]. AT, however, have a substantially lower 5-year survival rate of 25 to 69%, particularly due to their greater metastatic potential. Consequently, the malignant characteristics of bronchial carcinoids are likely due to its invasiveness and the intrinsic tumor stem cell population [1].

When advanced bronchial carcinoid tumors are not amenable to surgical resection a number of treatment modalities have emerged including chemotherapy, such as everolimus, targeting mTOR [1, 2]. However treatment resistance, relapse, and metastasis are currently still problematic [1, 2]. The inherent tumor-initiating cells (TIC; cancer stem cells) confer treatment resistance [3, 4]. TIC tumorigenic potential, capacity to repair DNA damage, their self-renewal property, and lack of functional regulation present in normal adult cells, suggest a need for targeted TIC therapy [5]. Thus treatment regimens that specifically target the TIC population are emerging, but are not yet well established [6].

Because TIC preferentially expand and survive in hypoxic niches, where hypoxia inducible factor-1α regulated carbonic anhydrase is induced, carbonic anhydrase inhibitors may be a plausible means for targeting tumor relevant pH homeostasis and eliminating TIC. Acetazolamide (AZ), a pan-carbonic anhydrase inhibitor is becoming recognized as a repurposed agent for treatment of cancer. AZ is currently primarily used for the treatment of glaucoma, epilepsy and altitude sickness [7]. Sulforaphane (SFN), a natural isothiocyanate with histone deacetylase inhibitor activity, can target multiple signaling pathways. SFN has been shown to be efficacious in eliminating TIC through the induction of the NF-kB, Shh, EMT and Wnt/beta-catenin pathways, as well as reducing the level of hypoxia inducible factor-1α [8–13]. In a previous study, we demonstrated that the combination of AZ + SFN significantly reduced clonogenic and invasive capacity, and induced growth inhibition of bronchial carcinoid and bladder cancer cell lines [11, 12]. Since AZ and SFN appear to show TIC targeting abilities [14, 15], the combination may be able to produce additive or synergistic anti-cancer effects.

In order to demonstrate the therapeutic efficacy of TIC-targeting treatments, appropriate models need to be utilized. Commonly used 2D monolayer cultured cells fail to recapitulate the tumor microenvironment due to the lack of cell-cell and cell-matrix interactions [16, 17]. In general, growth of primary bronchial carcinoid tumors in monolayer culture followed by intravenous injection to nude mice infrequently leads to tumor take [18]. In contrast, recent studies have shown that growing cells under spheroid promoting conditions reproduces the heterogeneity of tumor cells with expansion and enrichment of the TIC subpopulation [19–21]. Qiu et al., studying the small cell lung cancer cell line H446 grown under spheroid-promoting conditions and maintained for over 30 generations, demonstrated an enrichment of self-renewing TIC [22]. Spheroid grown cells display higher expression of TIC markers, ALDH1, Oct-4 and Nanog, compared to parental cells in monolayer culture [19, 23]. Also, 3D spheroid models exhibit increased clonogenicity and drug resistance in-vitro, and increased tumorigenicity in- vivo, in comparison to 2D monolayer grown cells [16].

Here we report that bronchial carcinoid cell lines H727 (TC phenotype) and H720 (AC phenotype) grown under spheroid-promoting conditions, in comparison to 2D monolayer cultures, were enriched for expression of the well characterized TIC stem cell markers, ALDH1, CD44, Oct-4, Sox-2 and Nanog, confirming presence of a TIC population. In addition, as compared to bronchial carcinoid monolayer grown cells, spheroids grown under stem cell conditions showed significantly increased tumorigenicity as xenografts. In pilot studies, we applied this approach to bronchial carcinoid samples from patients and developed a novel orthotopic model in lung where 3D spheroids co-cultured with fetal lung fibroblast cells demonstrated effective growth of tumors in-vivo. Finally, we evaluated the TIC targeting potential of AZ, SFN and AZ + SFN on H727 and H720 bronchial carcinoid cell lines in-vitro and in-vivo, in the different models. We now present evidence from pre-clinical models that SFN alone is highly effective in reducing tumor growth with further enhancement by the AZ + SFN combination.

## Methods

### Materials

RPMI-1640 medium and insulin was purchased from Sigma-Aldrich Canada Inc. (Oakville, ON, Canada) and phosphate buffer saline (PBS), penicillin streptomycin, and trypsin-EDTA (trypsin plus ethylenediaminetetraacetic acid) were obtained from Gibco (Carlsbad, CA, USA). Fetal bovine serum (FBS), human fibroblastic growth factor (hFGF), epidermal growth factor (hEGF), and B27 were purchased from PreproTech (Montreal, QC, Canada). The primary antibodies used were ALDH1 (ABCAM, Cambridge, MA, USA), CD44 (ABCAM Cambridge, MA, USA), Oct-4 (Cell Signaling, Danvers, MA, USA), Sox-2 (Gene Tex; Irvine, CA, USA) and Nanog (Cell Signaling, Danvers, MA, USA). Secondary antibodies obtained were anti-mouse (Fisher Scientific, Ottawa, ON, Canada), anti-rabbit (Invitrogen, Toronto, ON, Canada). Phosphate buffered saline (PBS) was purchased from Multicell (St. Bruno, QC, Canada). Methylcellulose based clonogenic medium as Methocult was acquired from StemCell Technologies (Vancouver, BC, Canada).

### Cell lines

The bronchial carcinoid cell lines NCI-H727 [H727] (typical carcinoid; ATCC® CRL-5815™) and NCI-H720 [H720] (atypical carcinoid; ATCC® CRL-5838™) used in this study were purchased from the American Type Culture Collection (ATCC). H727 and H720 cell lines were maintained in RPMI-1640 medium (Sigma-Aldrich Canada Inc., Oakville, ON, Canada) containing 10% fetal bovine serum and 0.5% penicillin- streptomycin. Cell lines were routinely monitored for mycoplasma using immunofluorescence detection, and maintenance of the characteristic phenotypes as described by ATCC. The cells were incubated at 37 °C with 5% CO2. Medium was replaced every other day by fresh medium for 7 days. Confluent cells were washed with PBS and then detached by 0.05% trypsin-EDTA (Gibco, Carlsbad, CA, USA). After centrifugation (1200 RPM at 4 min), cells were collected, counted and re-suspended in PBS at a concentration of 4 × 10^7^ cells/mL or medium. Both H727 and H720 cells were routinely checked for phenotypic fidelity. Cell lines were also routinely checked by DAPI fluorescence and morphologically for any signs of mycoplasma presence and effects on phenotype.

### 3D spheroid culture

H727 and H720 cell lines were cultured at a density of 10^6^ cells in 75 cm^2^ polymer-coated cell culture flasks to obtain 3D spheroids. The serum-free DMEM/F12 (1:1) medium used to culture the cells was supplemented with 30 ng/mL of hEGF and bFGF, 0.5% bovine serum albumin, 4 ng/ml of insulin and 0.03% B27. Twice a week the culture medium was replaced with fresh medium containing additional growth factors. After cell harvesting, trypsin was neutralized with complete medium and cells were gently centrifuged (1200 rpm for 4 min) and dissociated by trituration into single-cell suspensions. Culture and passage in the defined medium was then repeated until the development of third generation spheroids.

### Cell viability and drug cytotoxicity assays

Cell viability was determined by the trypan blue exclusion assay. After trypsinization cells were incubated with trypan blue (Multicell, Wisent Inc. St. Bruno, QC, Canada) for 10 min. The number of trypan blue positive per total cells per microscopic field (total of 4 fields per condition) were counted and calculated to obtain percent cell viability. To determine drug induced cytotoxicity 3 × 10^3^ parental cells and cells acquired from tumor spheroids of the H727 and H720 cell lines were plated into a 96-well plate. Then, a range of cisplatin doubling doses (2,4,8,16 μM) of cisplatin (CDDP; Sigma-Aldrich, Oakville, ON, Canada) was added to the cell medium. The cell viability after 3 days of treatment was evaluated by Trypan blue exclusion and AlamarBlue cytotoxicity assays. Baseline TIC relevant resistance to CDDP was determined by AlamarBlue cytotoxicity. IC50 values were calculated comparing parental cells with 3rd gen SP cells.

### Methylcellulose clonogenic assay

Clonogenic growth as spheroids was assessed for H727 and H720 for both parental and third-generation spheroids. Cells were seeded in 1% methylcellulose-based medium (MethoCult™ M3334, STEMCELL, Vancouver, BC, Canada) supplemented with RPMI-1640, 10% FBS and 1% antibiotics (100 μg/ml streptomycin and 100 IU/ml penicillin), cultured in 35 mm tissue culture dishes (Nalgene Nunc International, Rochester, NY, USA) and cultures incubated at 37 °C and 5% CO2. This culture step was repeated two more times and the number of colonies produced by the parental and third-generation spheroids were counted after 7 days using a grading dish on a phase contrast microscope (× 10). The degree of clonogenicity was assessed as the average number of colonies per dish for each cell line and the parental and third-generation spheroids.

### Limiting dilution analysis

Within a 96-well culture plate, 100 cells of the dissociated third-generation spheroids were plated in 150 μl of growth medium, obtaining a single cell per well. Every 5 days, 20 μl of the growth medium were added into each well, and after 14 days, the number of clonal tumor spheroids were analyzed and evaluated.

### Animals and in-vivo experiments

NOD/SCID female mice, four-to-six-weeks old, were acquired from The Hospital for Sick Children (SickKids) animal facility. Animal procedures were conducted according to the guidelines of the Lab Animal Services. The protocol for conducting in-vivo animal experiments was approved by the Animal Safety Committee of SickKids Research Institute. We used the technique previously described by Mokhtari et al. [14]. H727 and H720 parental and 3rd generation spheroid cells (3 × 10^4^) were injected into the subcutaneous inguinal fat pad of NOD/SCID mice (control parental versus spheroid groups). The indications for termination of experiment were tumor size exceeding 2 cm^2^ in diameter or signs of morbidity in animals. Tumor diameters were measured on a daily basis until termination. The long (D) and short diameters (d) were measured with calipers. Tumor volume (cm^3^) was calculated as V = 0.5 × D × d2. After euthanizing the mice by cervical dislocation technique, the tumors were resected, weighed and fixed in 10% neutral-buffered formalin at room temperature and processed for histopathology.

For the orthotopic model, surgical procedures were performed on NOD/SCID female mice as follows: before anesthesia induction, EMLA cream (lidocaine/ prilocaine cream) was applied topically on the midline of the chest wall to provide analgesia. Then, mask anesthesia was induced with Isoflurane (2%), and standard sterile surgical preparation and procedures were utilized. Body temperature was maintained with warming electrical pads. When the animal was no longer responsive to tail or hind paw pinch, the anterior chest wall was shaved. Thereafter, the animal was put on a surgical sterile blue sheet and was prepped with a swab soaked with Chlorhexidine three times and a minimal mid-line incision (1 cm) in the lower third of sternum made to gain access to the chest cavity. The level of anesthesia was monitored and supplemented as necessary using Isoflurane 2%. Animals’ breathing pattern was checked frequently during the procedure. Next, skin and underlying layers were carefully undermined to expose the right lung. Enough attention was paid to avoid injuries to heart or major vessels. A total volume of 2 μl containing either 1000 3rd generation spheroid cells (TC or AC) mixed with Matrigel (1:1) or 1000 bronchial carcinoid patient derived viable cells mixed with a supporting mesenchyme (fibroblasts, 1000 cells) and Matrigel (1:1), (BD Biosciences, San Diego, CA, USA) was injected by a pediatric insulin syringe (BD, Franklin Lakes, NJ, USA) into the lower third of the right lung. To perform the implantation precisely, a 45-degree angle was preferred for injection.

After injection, a careful observation was made to prevent any bleeding. The incision site (skin and deeper layers) was closed in a single layer by using an absorbable suture. For recovery and maintenance, animals, were placed in temperature controlled individual cages on warm surgical green towels for recovery. Recovery was signified by the alertness of the animal, normal mobility and breathing. After recovery, the animals were returned to animal housing in cages that were pre-labeled for control, AZ, SFN and AZ + SFN treatment. The candidate drugs were administrated to animals by intraperitoneal injection method for a period of 14 days.

During the post-operative period, animals were monitored daily for signs of pain and distress, as well as infection. Signs of pain and distress include lethargy, guarding in the affected area, restlessness, labored breathing, self-mutilation, lack of interest in food and water, lack of grooming, vocalization, difficulty urinating, weight loss, piloerection and aggressive or withdrawal behavior. Animals displaying distress received buprenorphine 0.1 mg/kg subcutaneously as an analgesic every 4–6 h initially until signs of distress were resolved. Animals displaying mild distress received acetaminophen in their water. Oral (tetracycline or enrofloxacin in drinking water) or injectable antibiotics (enrofloxacin 5-10 mg/kg q12h IM) were administered if indicated. Signs of infection at the incision site included erythema, discharge, abscess formation and wound dehiscence. Injectable analgesics and antibiotics were offered for greater control of dosage than oral, as these subjected the animals to additional stress. Oral analgesics and antibiotics were used only if required or if the animal was not consistently drinking. Any animals whose distress could not be resolved by the above procedures were then euthanized by cervical dislocation technique.

### Magnetic resonance imaging

**Fig. 1:**
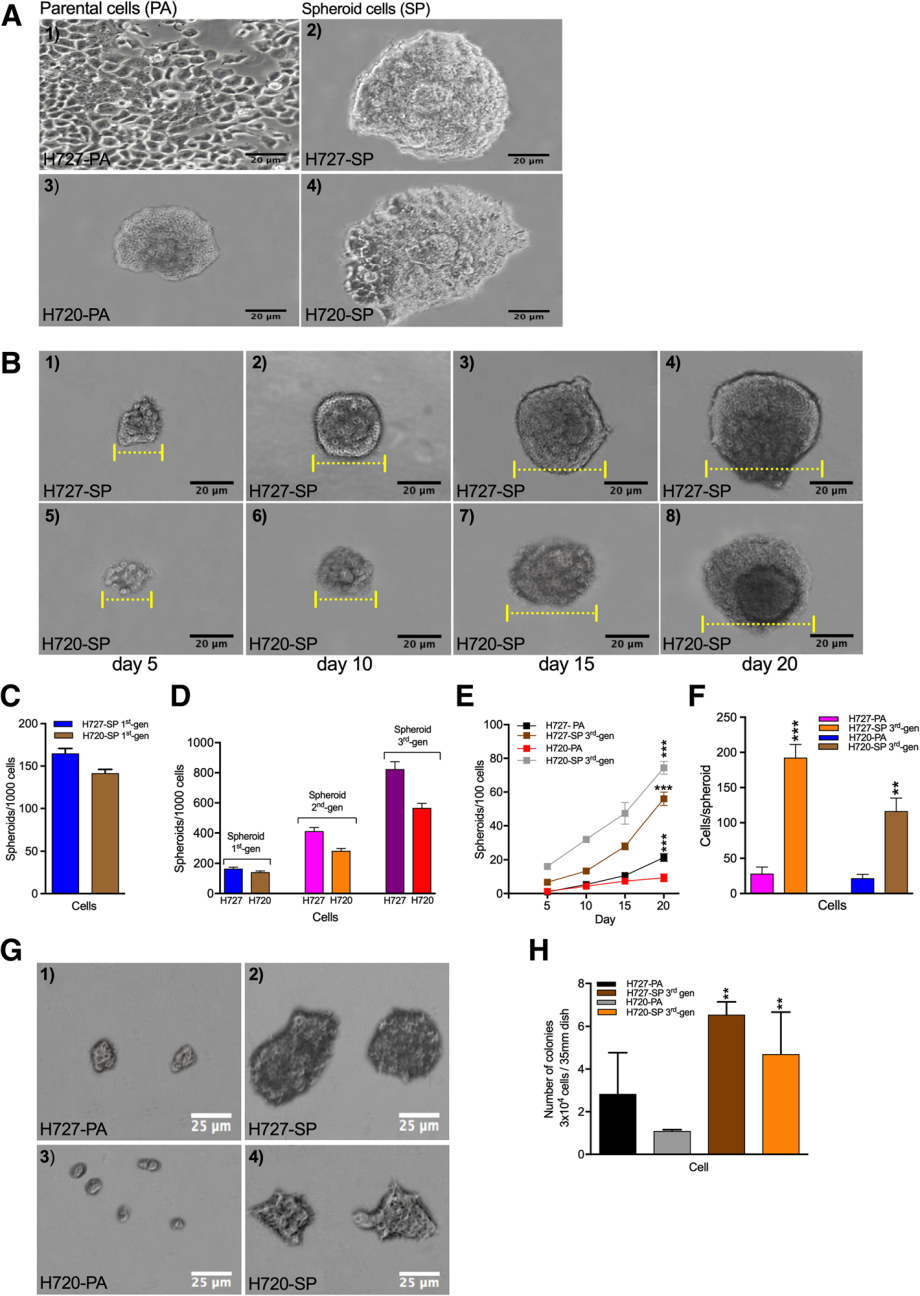
Bronchial carcinoid cell line growth assessed as monolayers and as spheroids with serially passaging and assayed by clonogenic assay show evidence of TIC. **a** Monolayer cultures of parental (PA) H727 and H720 cell lines were passaged under non-adherent stem cell culture conditions to permit spheroid growth (SP) visualized under phase microscopy. **b** Spheroids growing in methyl cellulose medium progressively expanded from an initial size of 200 to 300 cells per spheroid after 5 days to ~ 6 x the volume after 20 days. Both cell lines were able to form spheroids of different sizes and efficiencies. **c** Numbers of spheroids formed per 1000 cells seeded comparing H727 to H720. **d** First generation spheroids as in (**c**) were dissociated and replated to form 2nd and 3rd generation spheroids with a significant increase in spheroid numbers. **e** Spheroid forming ability evaluated over 20 days comparing PA cells with 3rd generation spheroids for both H727 and H720 starting from an initial seeding of 100 cells. **f** Spheroids formed from PA and 3rd generation spheroids were dissociated and cell numbers enumerated. **g** To assess clonogenic capacity 3 × 10^4^ cells from PA and 3rd generation spheroids of H727 and H720 were seeded in methylcellulose medium in 35 mm dishes and cultured for 7 days. Spheroids were visualized under phase microscopy. **h** Numbers of spheroids formed enumerated per dish in triplicates

Beginning on Day 7 after tumor cell inoculation, mice were imaged weekly in a 3-Tesla clinical magnetic resonance imaging (MRI) scanner, using an eight-channel wrist coil for signal detection (see Fig. 1). Mice were first induced on 2% isoflurane in pure oxygen (2 L/min flow rate) and then maintained on 1.5% isoflurane during imaging. Mice were placed prone within the coil, resting on top of a water-blanket maintained at 36 °C (HTP-1500, Adroit Medical Systems). High-resolution anatomical T2-weighted turbo spin-echo scans were acquired. The T2-weighted turbo spin-echo scan used a 2D acquisition with the following parameters: repetition time = 4000 ms, echo time = 75 ms, echo train length = 16, number of signal averages = 2, 100 mm field-of-view, twenty 1-mm thick slices, and 0.6 × 0.6 mm in-plane resolution. Tumors are detected as a hyperintense (i.e., bright) signal on T2-weighted images.

### Fluorescence and immunofluorescence staining methods

The uptake of riboflavin detected by autofluorescence has been shown to correlate with the presence of tumor stem cells [24, 25]. We used this method as published to detect the concentration of TIC in H727 and H720 derived spheroids. Parental cells (PA) of both H727 and H720 cell lines were cultured on cover slips in a 24- well plate and incubated at 37 °C and 5% CO2 for subsequent cell fixation. Third-generation H727 and H720 spheroids (SP) were transferred into eight 15 mL tubes. The cells were gently pipetted in fresh PBS and 80% cold methanol; the 24- well plate was fixed and immediately placed in a − 20 °C freezer for 10 min. The coverslips were transferred to the appropriate number of well plates and permeabilized with 0.3% Triton X-100 for 3 min. The cells were washed with PBS and blocked with 5% BSA in 0.1% PBS-Tween (PBST) for 30 min at room temperature. The primary antibodies Oct-4 (1:400), Sox-2 (1:400), Nanog (1:400), ALDH1 (1:150) and CD44 (1:100) made up in 5% BSA-PBST were transferred onto the cover slips and incubated at 4 °C overnight.

The cells were then washed with PBS and incubated with the appropriate fluorophore conjugated secondary antibody, anti-mouse and anti-rabbit made up in 5% BSA PBST, for 1 h at room temperature.

Sections were incubated with the secondary antibody alone as the negative control. The cells were washed with PBS again and incubated with DAPI nuclear counterstain for 10 min (1:10,000) at room temperature. Using a fluorescence microscope at × 10 and × 40 magnification (Nikon DXM1200 digital camera, 331 Norton Eclipse software version 6.1), the cells were visualized and fluorescence images were obtained.

### Immunohistochemistry

Parental and 3rd generation spheroids of both H727 and H720 xenografts were suspended in Matrigel and then processed into paraffin blocks. Sections were transferred onto microscope glass slides, deparaffinized through xylene and graded alcohols into water. Antigen retrieval was performed in 10 mM sodium citrate buffer (pH 6.0) with heating in a microwave oven for 10 min. The sections were cooled for 20 min at room temperature and then incubated in 3% hydrogen peroxide in water for 10 min to block endogenous peroxidase activity. The sections were washed 10x with deionized water for 5 min and incubated with the appropriate serum (10% goat serum and 10% horse serum in PBST) for 30 min to block non-specific binding. The sections were then incubated with the appropriate primary antibodies Oct-4 (1:500), Sox-2 (1:500), Nanog (1:10), ALDH1 (1:300) and CD44 (1:50) made up in 5% BSA PBST and incubated at 4 °C overnight. The sections were washed with PBS again (10 × 5 min) incubated in the appropriate secondary antibodies and then incubated for two minutes with DAB (3, 3′- diaminobenzidine; Vector Laboratories, Orton Southgate, Peterborough, United Kingdom). The sections were washed with deionized water again and counterstained with hematoxylin. Once counterstaining was complete, the slides were dehydrated and mounted.

### Western blot

The Western Blot protocol was performed as previously described (14). Briefly, 100μg of protein was loaded for H727 and H720 lysates. Oct-4, Sox-2, Nanog, CD44, ALDH1 (Cell Signaling Technology, Toronto, ON, Canada) antibodies were used at 1:1000 dilution. Secondary horseradish peroxidase conjugated antibodies (Jackson Immunoresearch, West Grove, PA, USA) were used at a dilution of 1:6000 and signal was detected with the Supersignal chemiluminescence detection system (Pierce Biotechnology, Rockford, IL, USA). GAPDH served as the loading control. Signals were quantified by densitometry relative to untreated values.

### Flow cytometry

FACS analysis for expression and quantification of ALDH1, Oct-4, Sox-2, Nanog and CD44 was performed as previously described [14]. Adherent cells and dissociated spheroid cells were obtained after trypsinization, washed and fixed with 4% paraformaldehyde in PBS. 10^5^ cells/ml were permeabilized with 0.1% Triton-X in PBS, washed twice with PBS and blocked with cold 5% BSA/PBS solution for 1 h at 4 °C for 15 min. Cells were immunostained with anti-ALDH1 and anti-CD44 conjugated to phycoerythrin (1:200) for 45 min. Cells were incubated overnight at 4 °C in primary antibody against Oct-4 (1:200), Sox-2 (1:200), and Nanog (1:200) in 5% BSA/PBS. Cells were subsequently washed three times with PBS and incubated with a chicken-anti-rabbit Alexa Fluor-488 (1:3500) or goat-anti-mouse R-Phycoerythrin (1:500) secondary antibody in 5% BSA/PBS, for 1 h in room temperature. After 3x washes with cold PBS, cells were resuspended in PBS solution containing 7-AAD (BD Pharmingen, San Jose, CA, USA) and then analyzed on a BD LSRII flow cytometric analyzer. Cells negative for 7-AAD were gated to exclude non-viable cells. Gating was determined from the negative trypsin controls.

### Statistical analysis

Each experiment was performed in three separate trials. The results were expressed as mean +/− SD. To test the statistical significance (*p* < 0.05) of each experiment, we used T-test and two-way ANOVA. The significance levels were *p* ≤ 0.05(*), *p* ≤ 0.01(**) and *p* ≤ 0.001(***).

## Results

### Bronchial carcinoid cell lines form spheroids in 3D culture and show evidence of a stem cell-like TIC progenitor population

To first evaluate bronchial carcinoids for presence of TIC characteristics the H727 and H720 cell lines were subjected to spheroid culture under stem cell culture conditions, and growth observed for 20 days. H727 and H720 cell lines were able to form spheroids (SP) that grew expansively in stem cell culture conditions when plated from monolayer culture (Fig. 1a). Spheroids showed a progressive increase in spheroid size and number starting from Day 5 until Day 20 (Fig. 1b and d). Furthermore, in methyl cellulose medium spheroids ranged from 200 to 300 cells per spheroid after 5 days of non-adherent culture and progressively enlarged over 20 days. Both cell lines robustly produced spheroids under the specified culture conditions.

In order to determine if 2nd and 3rd generation passaging of spheroids could increase the number of spheroids generated, indicative of selecting a more stem cell population, suspension grown spheroids were harvested, dissociated, and re-plated under spheroid culture conditions. The average number of spheroids generated per 1000 cells plated increased significantly by the 3rd passage for both H727 and H720. Figure 1c shows that for H727 spheroid numbers increased approximately 5 fold by the 3rd passage (160 ± 3 vs 412 ± 4 vs 824 ± 2). Similarly, spheroid numbers increased ~ 4 fold for H720 (141 ± 6 vs 283 ± 3 vs 566 ± 6). Thus culture in stem cell medium yielded an approximate several fold increase in the number of spheroids per 1000 cells plated from 1st to 3rd generation (3rd gen) (Fig. 1c and d).

With more limiting cell numbers, Fig. 1e compares the parental numbers with the 3rd gen SP numbers for 100 cells plated over culture time. The average number of spheroids per 100 cells increased by day 20 for H727-PA vs H727 3rd gen SP (9 vs 75) and for H720-PA versus H720 3rd gen SP (21 vs 56) suggested an increased number of clonogenic cells in the SP of both cell lines. As only 100 cells were seeded the resulting high number of spheroids also favored expansion of a significant fraction of clonogenic cells selected under stem cell culture conditions.

In contrast, in Fig. 1f a lower percentage of single cells derived from the bronchial carcinoid parental cells could regenerate spheroids when compared with single cells derived from 3rd-gen SP. Figure 1f further supports the notion of selection of a more proliferative stem cell population since the number of cells within the spheroids increased substantially from 1st to 3rd generation. As shown, after 20 days of culture, 65% of the single cells had generated new spheroids by day 5 and showed increased sphere-forming efficiency, spheroid size and cell numbers until day 15. Results suggest a significant percentage of single cells derived from 3rd gen SP are self-renewing cells and can be expanded and maintained in culture as tumor spheres. The average number of cells per spheroid for H727PA vs H727 3rd gen SP (28 vs 193) and for H720PA vs H720 3rd gen SP (22 vs 116) represents a 5–7 fold difference and supports the idea of a more concentrated fraction of clonogenic cells in SP. Therefore spheroid forming bronchial cells show increased clonogenic potential.

As further direct evidence of clonogenic potential, we compared the number of colonies produced by 3 × 10^4^ cells for parental (PA) versus 3rd gen SP using a shorter 7-day assay. Figure 1g and h present the results of methylcellulose assay and quantification of 3rd gen SP for H727 and H720. Clonogenic potential for H727 PA versus 3rd gen SP increased from 2 ± 0.85 to 6 ± 0.55 and for H720PA vs 3rd gen SP from 1 ± 0.15 to 4 ± 0.75. Thus Fig. 1g and h showed not only a significant increase in colony formation but also colony size by the 3rd gen SP cells indicating increased growth potential. Altogether spheroid growth appears to favor expansion of a self-renewing TIC population in both TC and AC cells. The results suggest that approximately 50% of single cells from spheroids after serially passaging formed spheres and were able to be maintained in stem cell culture conditions.

### Bronchial carcinoid cell line derived spheroids exhibit characteristics of stem cells and show significantly increased expression of stemness markers

**Fig. 2:**
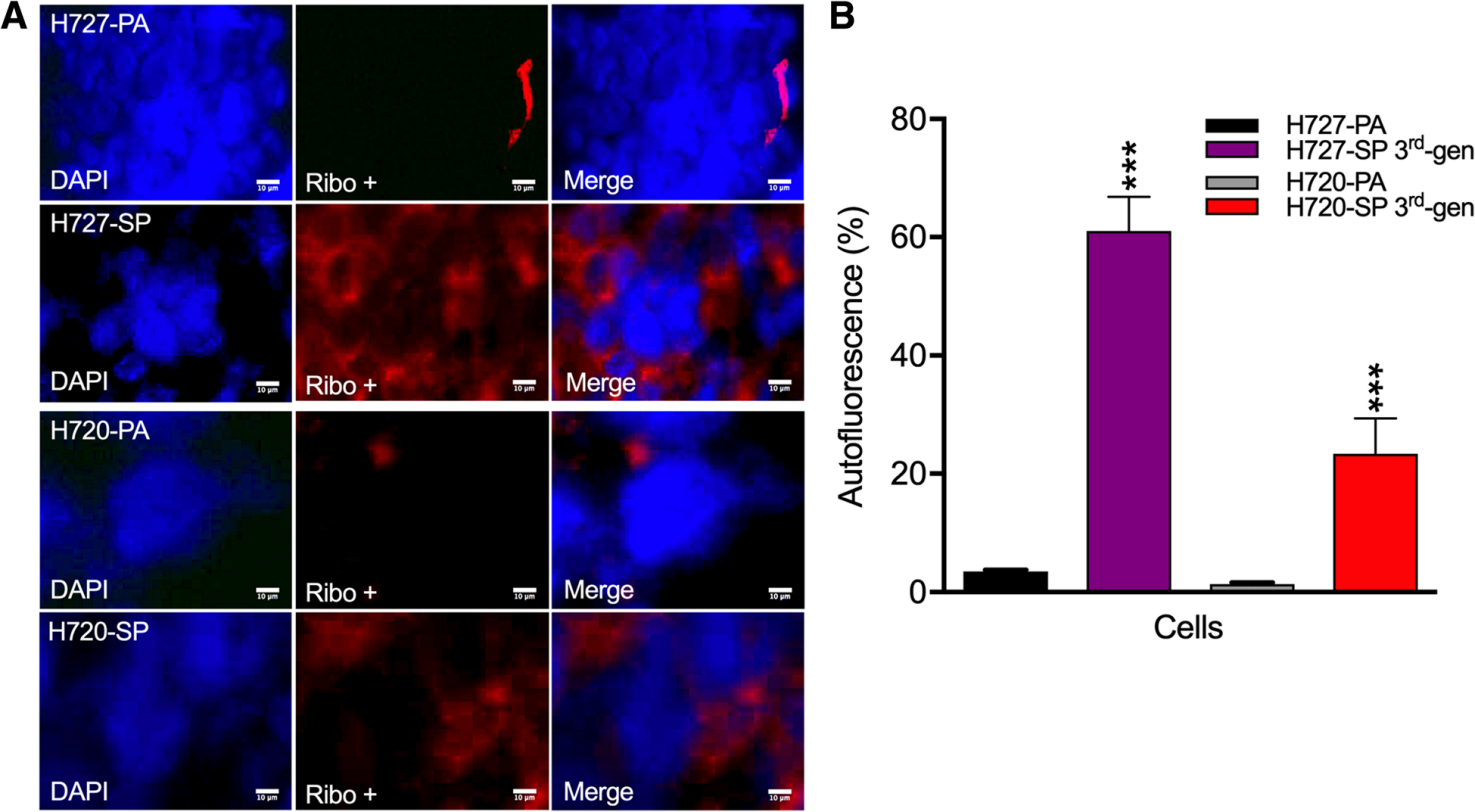
Riboflavin significantly accumulates in bronchial carcinoid spheroids. **a** Monolayer grown H727 and H720 cell lines and 3rd generation cultured spheroids were incubated with riboflavin, a marker for stemness, and uptake visualized by fluorescence microscopy. Autofluorescence images were captured. **b** Autofluorescence indicating riboflavin uptake mainly in the nucleus was quantified and shown as a percentage. Experiments conducted in triplicates

To initially characterize the TIC phenotype of cells within the spheroids, we exploited the reported finding that stem cells show a remarkable uptake of riboflavin [24, 25]. Thus, spheroids were incubated in riboflavin and showed strong autofluorescence that increased significantly from the very low levels in parental cells compared to the 3rd gen SP (Fig. 2a and b). Whereas 3 ± 0.5% H727-PA cells were labeled 60 ± 0.15% of H727-SP cells were labeled, a remarkable 20-fold difference. Similarly for H720-PA cells versus H720-SP an ~20fold difference in labeling was noted (1 ± 0.3% vs 23 ± 0.35%). Interestingly, the TC line H727 took up more riboflavin than the AC line H720 by nearly 3-fold difference (Fig. 2b). However, it should be noted that H720-PA cells were minimally labeled as compared to the H720-SP cells suggesting overall a very small proportion of TIC in monolayer grown cells. Thus, both increasing spheroid forming potential and riboflavin uptake supported the presence of a more concentrated TIC population in SP.

**Fig. 3:**
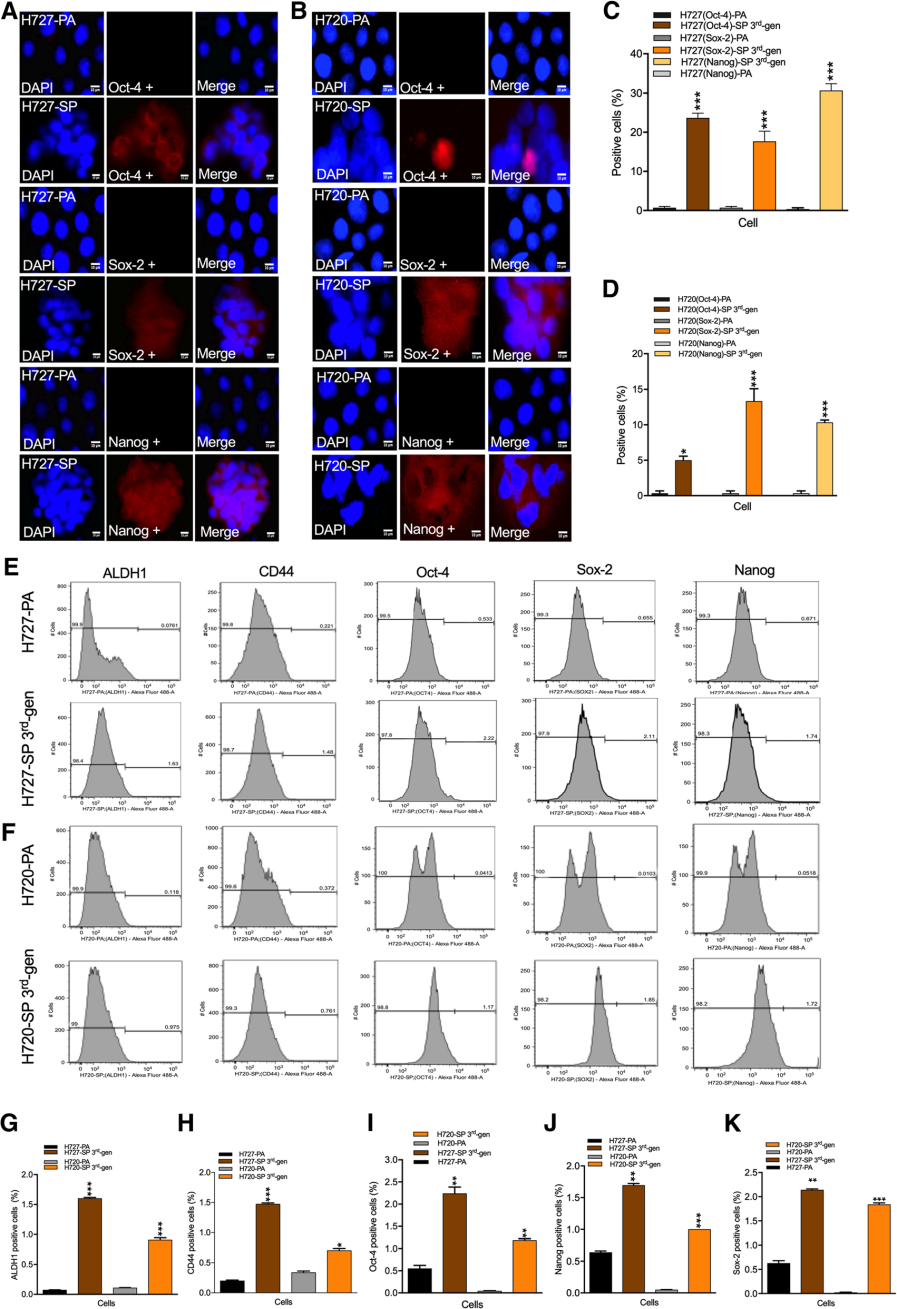
Expression of stem cell markers are increased in spheroid forming bronchial carcinoid cells. **a**&**b** Parental (PA) and 3rd generation spheroid cells (SP) were immunofluorescently labeled for expression of stemness markers, Oct-4, Sox-2 and Nanog visualized as in (**a**) and percentage positive cells quantified as in (**c**). Similarly, H720 PA and SP cells were fluorescently labeled and number of positive cells quantified (B&D). H727 and H720 PA and 3rd generation SP cells were immunolabeled for expression of ALDH1, CD44, Oct-4, Sox-2 and Nanog and the fraction of positively labeled cells assessed by FACS analysis (**e**&**f**). The number of positive cells in PA versus 3rd generation SP for all the markers as in (**e**&**f**) were quantified and expressed as a percentage fraction (**g**-**k**)

The in vitro results suggested presence of a significant TIC fraction selected under stem cell culture conditions. To reveal the stemness characteristics in the bronchial carcinoid cell lines, we used a panel of well characterized stemness markers and compared parental cells with 3rd gen SP cells by immunofluorescence labeling (Figs. 3a-d for H727; and H720) and by FACs analysis (Fig. 3e-k). Quantification of the immunofluorescence labeling revealed increased expression of stem cell markers in SP. In H727-PA versus H727-SP Oct-4 expression increased from 1 ± 0.15% to 23 ± 0.60%, Sox-2 from 0 ± 0.30% to 17 ± 0.56%, and Nanog from 0 ± 0.33% to 30 ± 0.68%. This constituted approximately a 20-fold increase in stemness marker expression corroborating the clonogenic observations.

In H720-PA versus H720-SP Oct-4 expression increased from 1 ± 0.16% to 5 ± 0.5%, Sox-2 from 1 ± 0.03% to 13 ± 0.10%, and Nanog from 1 ± 0.05% to 10 ± 0.11%. This lesser fold increase in stemness expression as compared to H727 was nevertheless still significant and in line with the clonogenic results.

Examining stemness expression by quantitative FACS analysis still showed increased expression of stem cell markers in SP as follows: H727-PA cells: ALDH1 (0 ± 0.07%), CD44 (0 ± 0.20%), Oct-4 (0 ± 0.55%), Sox-2 (0 ± 0.62%) and Nanog (0 ± 0.64%), compared to H727-SP cells: ALDH1 (1 ± 0.06%), CD44 (1 ± 0.47%), Oct-4 (2 ± 0.26%), Sox-2 (2 ± 0.14%) and Nanog (1 ± 0.69%). H720-PA cells: ALDH1 (0 ± 0.11%), CD44 (0 ± 0.34%), Oct-4 (0 ± 0.04%), Sox-2 (0 ± 0.02%) and Nanog (0 ± 0.02%) compared to H720-SP cells: ALDH1 (0 ± 0.91%), CD44 (0 ± 0.70%), Oct-4 (1 ± 0.19%), Sox-2 (1 ± 0.84%) and Nanog (1 ± 0.11%). The discrepancy with immunofluorescence labeling could simply be a technical issue because of different preparation conditions. Overall, immunolabeling results for Oct-4, Sox-2 and Nanog showed a marked increase in the 3rd gen SP cells and further supported by FACs analysis.

### Bronchial carcinoid spheroid cells show the stem cell feature of increased drug resistance

**Fig. 4:**
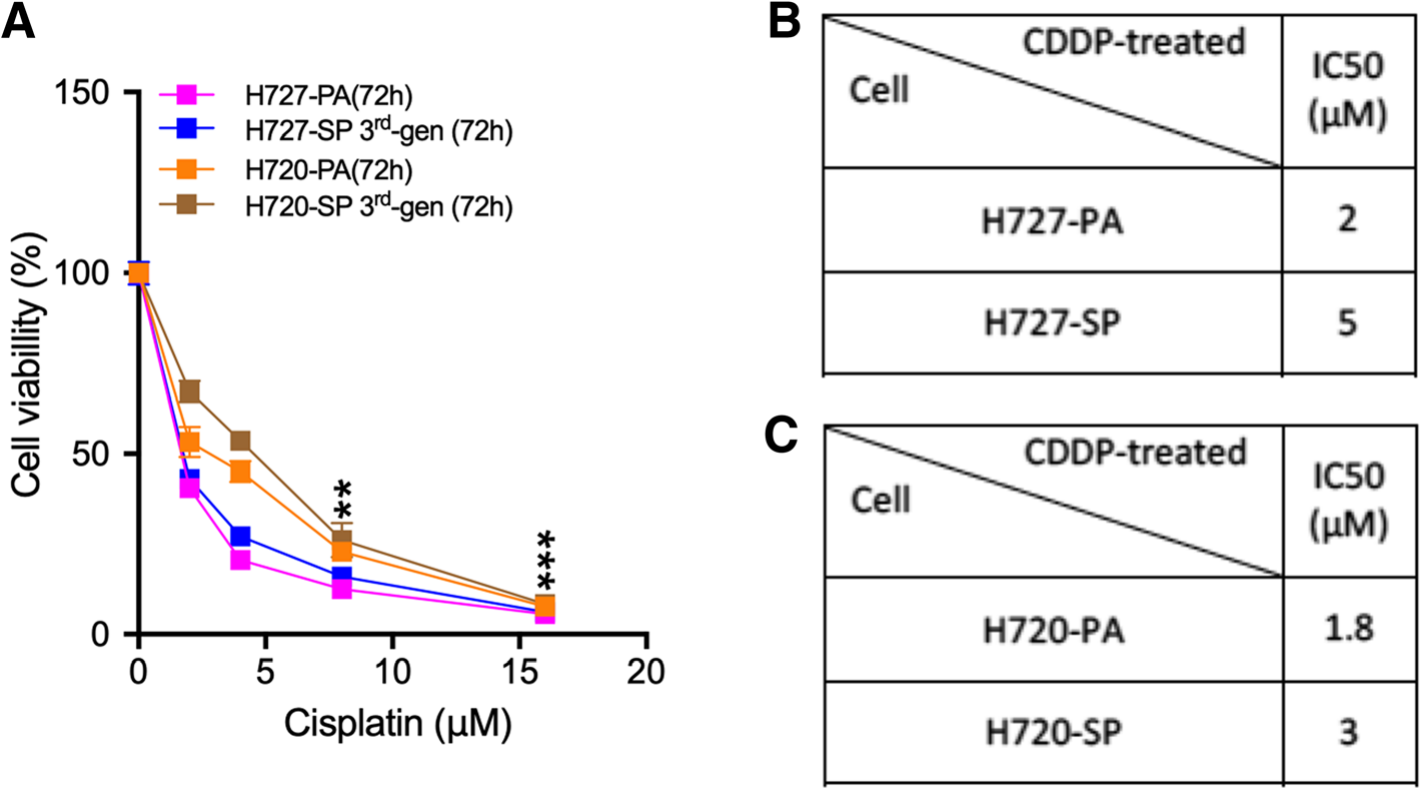
Spheroid cells demonstrate increased drug resistance. (**a**) H727 and H720 PA and 3rd generation SP cells were treated with increasing doubling doses in a range (0-16 μM) of cisplatin as shown and cytotoxicity assayed with AlamarBlue. Percentage cell viability was measured. **b**&**c** IC50 values were calculated for the response of H727 and H720 PA versus SP to cisplatin and presented in Tables

It is well demonstrated that stem cells show increased drug resistance compared to the non-stem cell fraction in cell lines [26]. This can be readily tested by resistance to cisplatin which is used in the treatment of bronchial carcinoids [27]. Figure 4a shows results of determining loss of viability after exposure to varying low μM concentrations of cisplatin. Figure 4b and c present the IC50 values comparing cisplatin toxicity on parental versus the 3rd generation spheroid cells. Data show that drug resistance increases by 26 ± 0.87% in H727-SP and 22 ± 0.15% in H720-SP, compared to untreated PA controls. Both cell lines were sensitive to the cytotoxicity of cisplatin, however significantly less so when grown as SP.

### Bronchial carcinoid spheroid cells show increased tumorigenicity when xenografted into immunocompromised mice

**Fig. 5:**
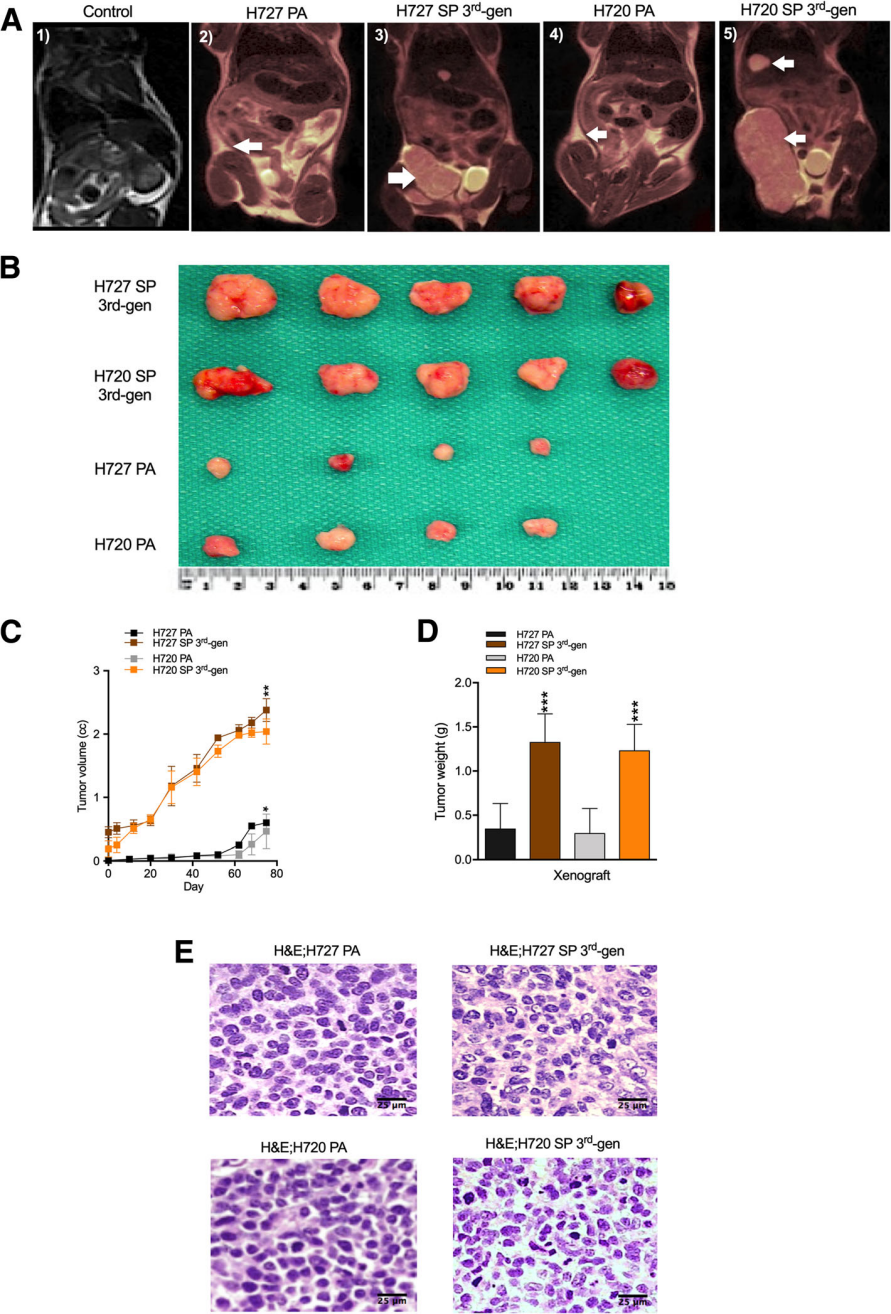
Spheroid cells demonstrate increased tumorigenic potential. **a** Magnetic resonance imaging (MRI) was performed on the fifth inguinal mammary fat pad of mice 10 days post-implantation of PA monolayer cells and 3rd generation spheroid (SP) bronchial carcinoid cells (H727 and H720; 3 × 10^4^ cells, *n* = 5) implantation; **b** Examination of extirpated tumors (*n* = 5) by gross morphology; **c** Tumor volumes measured over 80 days given in cc calculated from caliper measurements; **d** Tumor weights measured after extirpation given in grams. **e** H&E histology of the PA and SP cell derived xenografts

Given that cancer stem cells possess a greater tumorigenic potential than the non-stem cell fraction, we next determined if the presence of a substantial TIC fraction in 3rd gen SP would show significantly increased tumorigenic potential than the parental cell lines when xenografted into immunocompromised mice. Parental as well as 3rd gen SP cells were injected into NOD/SCID mice (3 × 10^4^ cells injected per mouse; *n* = 5). Results in Fig. 5a-d provide MRI evidence of xenograft growth. Tumor growth measurements of volume and weight were determined over the in-vivo 80-day growth period confirming robust tumor cell expansion from < 0.4 cc to ~ 2.5 cc for the 3rd gen SP cells and < 0.1 cc to ~ 0.3 cc for PA cells. While both H727 and H720 parental cell lines produced tumors in-vivo when heterotransplanted, the 3rd gen SP cells produced significantly larger tumors (~ 6-fold increase) in the same time period exceeding those produced by parental tumors in volume and weight (Fig. 5b-d). Note also the exponential growth of spheroid cell produced tumors versus parental cells, strikingly evident at 50 days. Furthermore, growth morphology of the resulting tumors suggested an increased vascularization of the SP generated xenografts. Histological analysis of the resulting tumors (Fig. 5e) did not reveal any overt tumor cell morphological differences except for features of vascularization as also noted grossly. The results are consistent with in-vitro data that 3rd generation spheroids were enriched in TIC leading to aggressive growth of xenografts.

### Acetazolamide (AZ) and sulforaphane (SFN) are effective anti-tumor agents against the TIC component in bronchial carcinoid 3rd generation spheroids

**Fig. 6:**
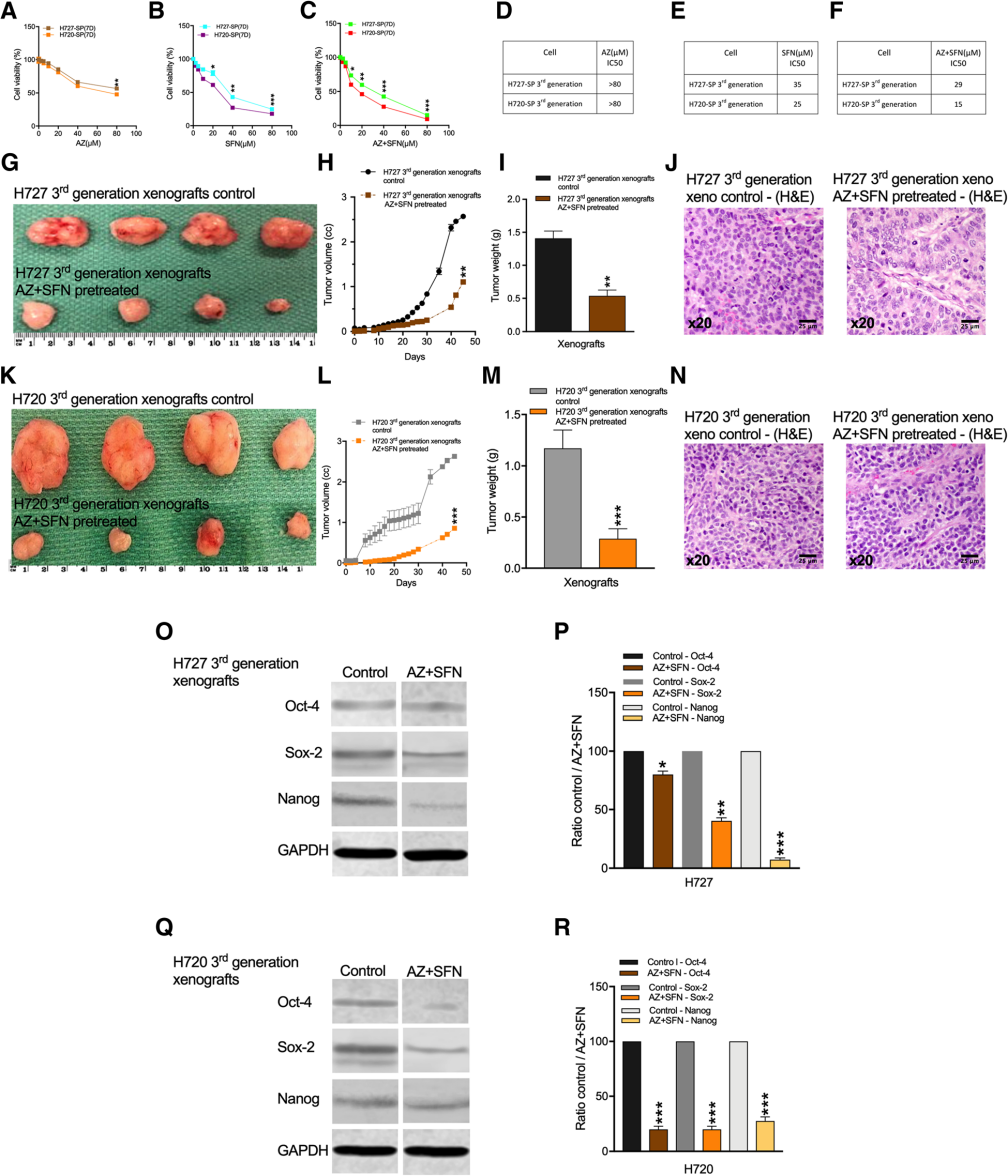
Growth of H727 and H720 derived spheroids was inhibited by AZ, SFN and AZ + SFN treatments. **a,c**&**e** Represent the AlamarBlue assay and **b, d**&**f** IC50 values of 3rd generation spheroid (SP) cells of H727 and H720 after 7 days treatment with AZ, SFN and the AZ + SFN combination (0-40 μM). **g**-**j** To further evaluate the anti-tumor effect of the AZ + SFN combination 3rd generation H727 xenograft cells were pre-treated with AZ + SFN, xenografted as before and tumor growth monitored over 45 days (*n* = 4). **g** showing gross morphology; H and I tumor volumes calculated after caliper measurements and weights measured after resection. J) H&E histology of the resected tumors. **k**-**n** As above H720 3rd generation xenograft cells were pretreated with AZ + SFN and resulting xenografts (*n* = 4) assessed by gross morphology (**k**), volumes over 45 days (**l**), weights of resected tumors (**m**) and histology of resected tumors (**n**). **o**-**r** Western blot analysis was performed for Oct-4, Sox-2, and Nanog relative to the loading control (GAPDH) comparing untreated with AZ + SFN pretreated H727 and H720 xenografts. Signals quantified by densitometry relative to untreated control

We have previously reported that AZ and SFN, and especially the AZ + SFN combination can potently inhibit bronchial carcinoid growth and survival demonstrated in-vitro and in-vivo [11]. This previous study was conducted only on the parent cell lines. Here we asked if these agents could effectively target the TIC component in 3rd generation spheroids. Spheroids were treated with increasing doses of AZ, SFN and AZ + SFN and the IC50 values were calculated from the AlamarBlue assay. Whereas AZ alone significantly inhibited proliferation at 80 μM, SFN treatment caused a ~ 60–75% reduction in proliferation at 40 μM and the AZ + SFN combination further reduced proliferation to < 60–75% at 40 μM (6A-C). IC50 values showed a modest potency for AZ alone and a several fold increase in potency for SFN versus AZ while the combination further increased the potency of SFN for H727 (35 μM vs 29 μM) and even greater for H720 (25 μM vs 15 μM), (Fig. 6d-f). Thus Fig. 6a-f show a dose dependent decrease in H727 and H720 viability by AZ, SFN and the AZ + SFN combination. SFN was more potent that AZ as shown by the IC50 values in the 20 μM to 80 μM range, while the AZ + SFN combination showed the lowest IC50 values. Interestingly, the AT bronchial carcinoid variant H720 was more sensitive to the effect of the combination.

The above results indicate that stem cell growth conditions in suspension culture selects for and promotes expansion of the TIC fraction in the parental lines. We re-examined the idea that AZ + SFN could effectively target the TIC fraction resident within the cell lines that was selectively expanded into 3rd gen SP.

In xenograft studies AZ + SFN caused a significant reduction in growth of H727 3rd gen SP tumor volumes (45 ± 1.1%; *p* = 0.01) and tumor weights (0.5 ± 0.038%; *p* = 0.01) (Fig. 6g-i). Again note the marked reduction in gross vascularization. H&E histology (Fig. 6j) suggested a reorganization of tumor cells into columnar differentiation around blood vessels. For H720 3rd gen SP tumors a reduction in volumes (> 65 ± 0.85%; *p* = 0.001) and tumor weights (0.2 ± 0.08%; *p* = 0.001) was noted after 45 days compared to xenografts generated from parental cells (Fig. 6k-m). Interestingly, the combination was more effective against H720, the bronchial carcinoid AT phenotype. Thus the results in Fig. 6 establish the increased tumorigenic potential of 3rd gen SP cells and sustained growth suppression by AZ + SFN. The strong growth inhibition by AZ + SFN would be expected to target the TIC fraction.

The overall 3–4 fold reduction in tumor volumes and histology, suggesting a possible induction of differentiation, favoring alterations in the stemness component. We therefore examined tumors for expression of stemness markers. Western blot analysis of Oct-4, Sox-2, and Nanog relative to the loading control (GAPDH) was performed on the control untreated and AZ + SFN pretreated xenografts. Figure 6o-p show that after AZ+ SFN treatment of H727 3rd gen xenograft tumors significantly lower expressions of stemness markers Oct-4 and especially Sox-2 and Nanog were found. Figure 6q and r present the results for H720 3rd gen SP derived xenograft tumors with marked reductions in expression of all three stemness markers Oct-4, Sox-2 and Nanog. Data show that expression of stem cell markers Oct-4, Sox-2 and Nanog decreased in H727 3rd gen SP derived xenografts (1.25, 2.5 and 13.6-fold) and those from H720 3rd gen SP cells (5, 5 and 3.6-fold). The marked reduction in stemness markers for H720 aligns well with the greater reduction in tumor growth after pre-treatment with AZ + SFN. Thus overall AZ + SFN pre-treatment significantly reduced the in-vivo tumor growth potential of both H727 and H720 3rd generation spheroid cells. The data also indicate that the less differentiated AT variant may be more stem cell like in its phenotype and that AZ + SFN can effectively reduce expression of all three stemness markers. Thus, the in-vivo xenograft model demonstrates the efficacy of the combination therapy against the TIC of the two bronchial carcinoid cell lines. It also demonstrates that exploiting 3rd generation spheroid formation was an effective means to assess the therapeutic potential of the AZ + SFN combination.

### Combination therapy targets the bronchial carcinoid TIC population within the orthotopic lung microenvironment

**Fig. 7:**
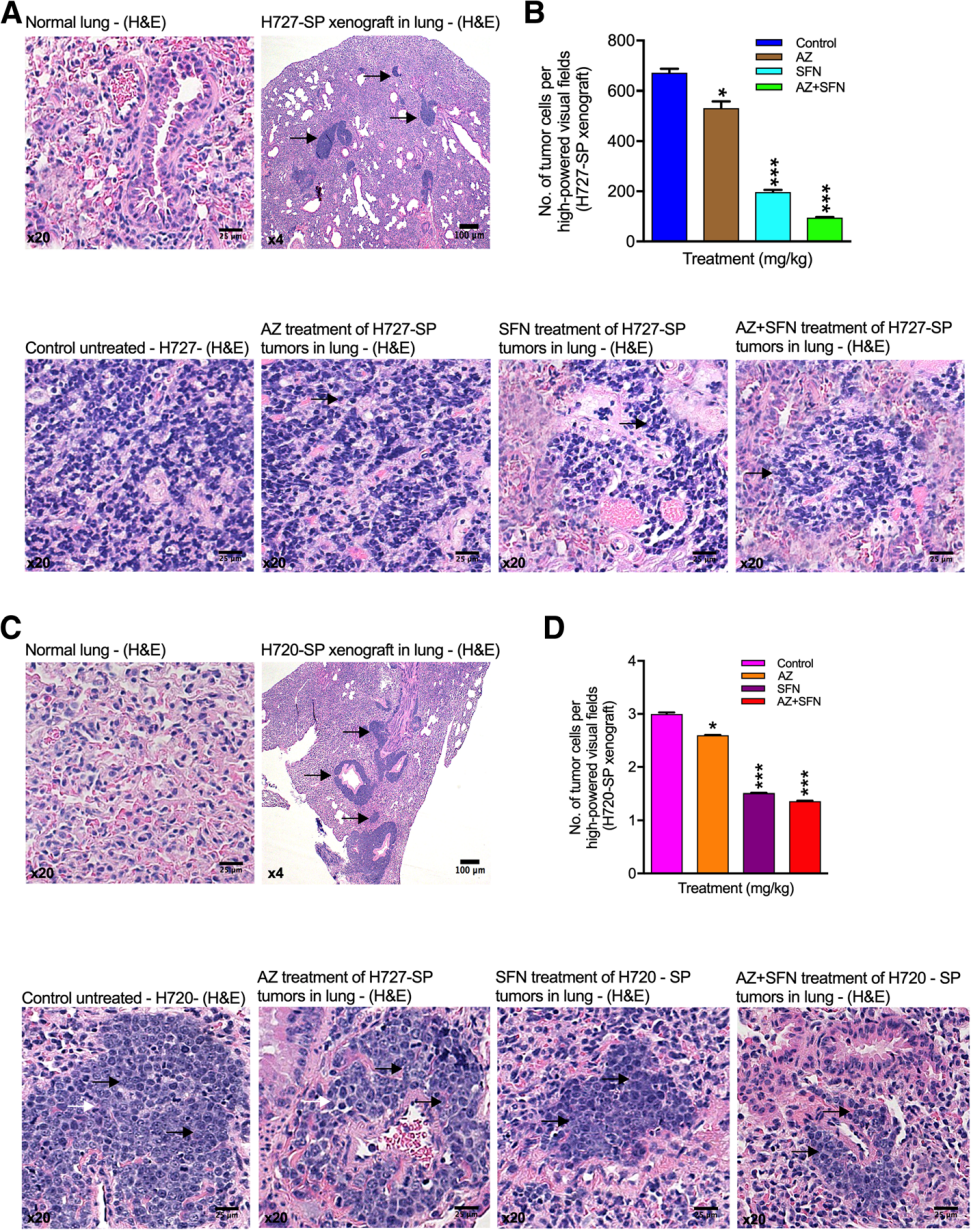
Spheroids developed from H727 and H720 were orthotopically heterotransplanted into mice lungs and formed foci of dense tumor cells. **a** H&E histology of normal lung versus H727 3rd generation SP cells orthotopically xenografted in mouse lungs and tumors allowed to developed over days. Arrows point to tumor masses in lung parenchyma. **b** Lung xenografts analyzed after treatments with AZ, SFN and AZ + SFN in 40 mg/Kg were assessed for number of tumor cells per high-powered field. Below: H&E histology of tumor fields in these treated mice comparing untreated, AZ, SFN and AZ + SFN treatments. **c** A similar experiment as in A&B was performed for H720 3rd generation SP cells orthotopically xenografted into mouse lung. **c** histology; **d** number of tumor cells per high powered field; Below: H&E histology on H720 3rd generation SP generated orthotopic tumors comparing control with AZ, SFN and AZ + SFN treatments

Although the subcutaneous tumorigenicity model is an informative pre-clinical model, given that bronchial carcinoids arise and expand within the environment of lung it was relevant to determine if the 3rd generation spheroid cells of H727 and H720 possessed an ability to form tumors in lung, this being the pathognomonic orthotopic model. Vigorous growth in lung might be expected based on the evidence of TIC in spheroids as these would have greater tumorigenic potential. Furthermore, this model would allow us to mimic patient therapy when evaluating the potential of our anti-tumor therapy. Therefore we established the lung orthotopic model for both H727 and H720 bronchial carcinoid cell lines and tested the in-vivo effect of AZ (40 mg/kg), SFN (40 mg/kg) and AZ+ SFN. Figure 7a shows the results of orthotopic tumor growth in lung for H727. Figure 7b shows that AZ, SFN and AZ + SFN significantly reduced the number of tumor cells in lung xenografts relative to untreated controls with a 6-fold reduction after AZ + SFN ((AZ: 532, SFN: 197 and AZ + SFN: 95). Notably SFN treatment alone was highly effective but further potentiated by addition of AZ. H&E histology confirmed the anti-tumor effects with significant loss of tumor cells after SFN and AZ + SFN treatments. Replacement of tumor cells with a stromal component was noted.

In the case of H720, H&E histology showed robust growth of orthotopic tumor nodules (Fig. 7c) and then a significant loss of tumor cells after treatments with AZ, SFN and AZ + SFN (AZ: 330, SFN: 125 and AZ + SFN: 45) (Fig. 7d). The fold reduction was not as great as that for H727, however, addition of AZ to SFN still further potentiated the reduction. H&E histology of the resulting xenografts before and after treatments revealed that AZ slightly disrupted tumor nodule architecture whereas SFN and AZ + SFN caused overt alterations and an increase in stromal component. We inferred that SFN and AZ + SFN treatments resulted in either a fibrotic like response or dispersion of the tumor foci suggesting possible phenotypic changes. Thus SP cell-generated bronchial carcinoid orthotopic xenografts demonstrate greatest inhibition of tumor cell growth by AZ + SFN treatment. Although histological analysis revealed that AZ alone did somewhat reduce tumor cell density, SFN was significantly more effective with a further reduction in tumor cell density by AZ + SFN combination. The significant reduction in tumor cell number and replacement with fibrous tissue suggested both growth inhibition and ultimately tumor cell death. This was more evident in H727 tumors while histology of H720 tumors suggested tumor cell dispersion and a greater change in phenotype. Tumor morphological differences were still evident compared with intact surrounding normal lung. The orthotopic lung model did highlight that bronchial carcinoid tumors growing in their site of origin were still sensitive to the inhibitory effects of AZ + SFN supporting the translational potential of these agents.

**Fig. 8:**
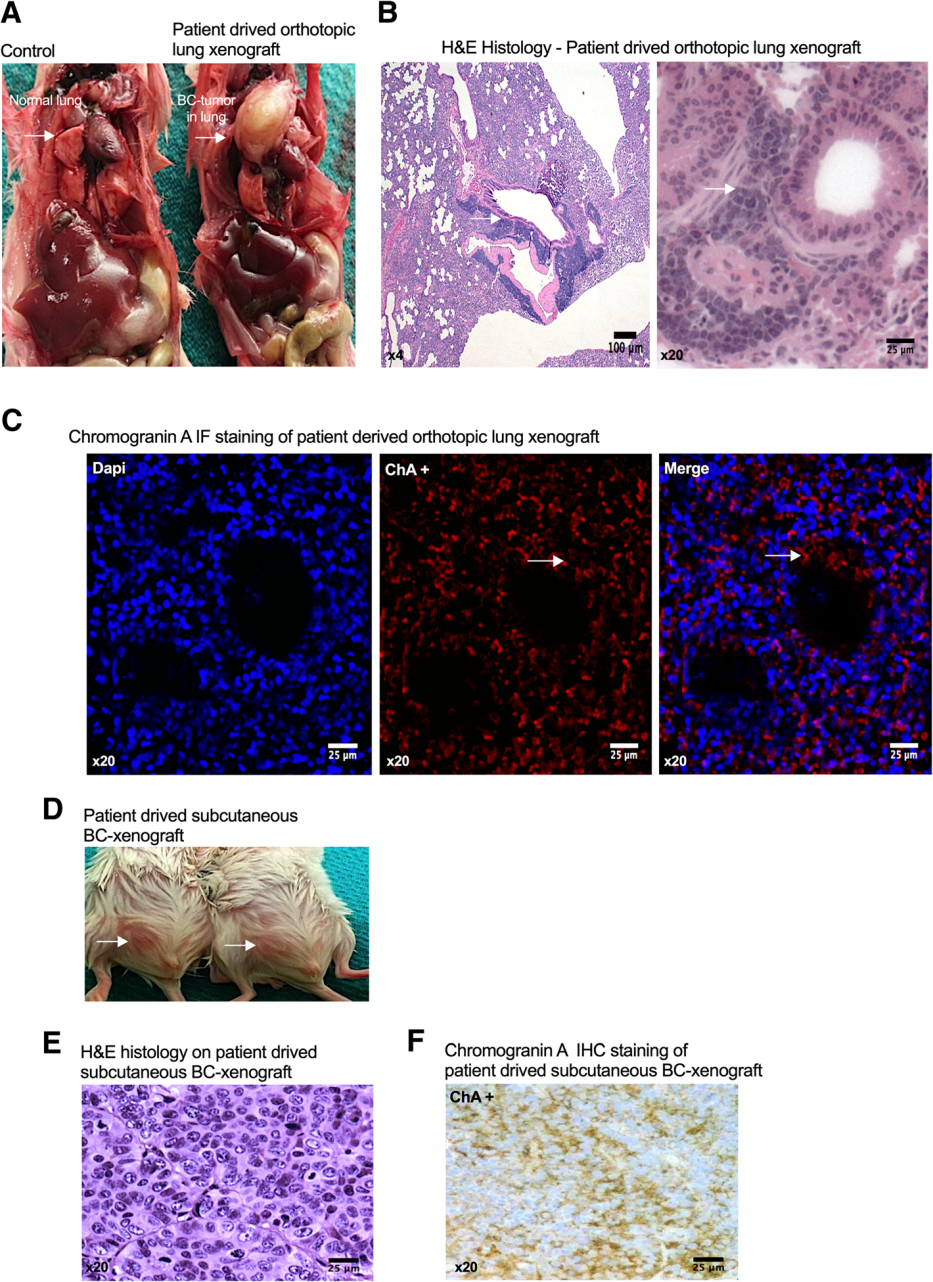
Bronchial carcinoid tumor patient-derived spheroids show high tumorigenic potential both orthotopically and subcutaneously. **a** Shows the gross morphology of normal lung (left) and the patient-derived orthotopic tumor (right) in lung (arrows); **b** H&E histology at lower and higher magnifications of the patient-derived tumor showing typical BC morphology and dense cellularity; **c** Immunofluorescence labeling for Chromogranin A (ChA) in patient derived orthotopic lung xenograft. DAPI counterstain for labeling of cell nuclei. **d** Gross morphology of patient derived subcutaneous xenografts (arrows) in mouse subinguinal region. **e** H&E histology of the resected subcutaneous xenograft; **f** Immunohistochemical staining for Chromogranin A in subcutaneous xenografts

Lastly, we asked if direct orthotopic implantation of fresh patient bronchial carcinoid cells could form tumors in lung. We obtained a small fresh sample of resected primary tumor tissue of a verified lung bronchial carcinoid from a post-menopausal female patient, 64 years (Dr. R. Govindan, St. Louis; IRB approved) sent in serum and antibiotic containing medium. The tissue was triturated and set up as a primary culture in the medium similar to the neuroendocrine cell lines and observed for 7 days. Thereafter attached and floating cells were pooled, trypsinized and switched to stem cell medium and spheroid cultures established as described above. We then developed a Patient-Derived Xenograft model (PDX) based on further passaging to obtain the 3rd gen SP and added a fibroblastic mesenchyme (human fetal lung fibroblasts; ~ 1:3 ratio) to support the spheroids for xenografting. We found that these complex spheroids readily formed tumors in immunocompromised mouse lungs (Fig. 8a and b) with a typical bronchial carcinoid phenotype as verified histologically and by expression of chromogranin A, a definitive neuroendocrine marker (Fig. 8c). The lung tumors were compared with the same cells implanted in the inguinal (subcutaneous) position, which also formed histologically typical bronchial carcinoid tumors (Fig. 8d and e) and similarly expressed chromogranin A (Fig. 8f).

## Discussion

Developing appropriate in-vivo bronchial carcinoid models are critical for evaluating the therapeutic potential of bronchial carcinoid targeting drugs. Currently, there are lung tumor models for squamous cell lung cancer and small cell lung cancer, but there is a lack of any bronchial carcinoid tumor models [28, 29]. As bronchial carcinoids are more indolent, slow growing, highly differentiated, and of low tumorigenic potential, it has made it difficult to develop xenograft models to enable the study of bronchial carcinoid pathology and develop means for drug development [30]. However, considering the strong in-vitro growth of H720 and H727, along with the aggressiveness of atypical bronchial carcinoids, we posited that culture of these bronchial carcinoid lines in serum-free stem cell conditions would form 3D spheroids (SP) that are known to recapitulate the tumor phenotype allowing for concentration of TIC as could be verified by expression of stemness markers. We had previously reported that the novel AZ + SFN combination was effective in reducing the tumorigenic potential of H727 and H720 bronchial carcinoid parental cells [11].

Here, we demonstrate that H727 and H720 cell lines grown in serum-free stem cell supporting medium contain a significantly higher proportion of the TIC population than parental monolayer cells. By passaging H727 and H720 cell line generated spheroids in the stem cell serum-free medium until the 3rd generation there was a substantial increase in spheroid forming ability. As a result, 3rd gen SP contained a higher TIC population because of the enhanced self-renewal potential. At equivalent seeding numbers the 3rd generation SP cells formed a significantly larger number and increased size of spheroids than parental cells. We were able to demonstrate the significantly enhanced tumorigenicity of these 3rd gen SP cells. Here we show that 3rd gen SP cells xenografted into the subcutaneous position yielded rapidly expanding tumors from an approximate 2 log enrichment of successful engraftment in subcutaneous tissue. In addition, 100% of the 3rd gen SP cell preparations developed tumors at a much shorter doubling time than parental cells, confirming the high proportion of TIC in spheroids. Increase in tumorigenic potential was shown by serial heterotransplantation. We further demonstrate that the AZ + SFN combination was highly effective in inhibiting clonogenicity and tumorigenicity of the 3rd gen SP cells. Concomitantly, spheroids and SP derived tumors showed a significant reduction in expression of the stemness markers reinforcing the idea of TIC targeting.

There are currently no definitive stem cell markers for bronchial carcinoid tumors. However, there have been some studies showing elevated expression of markers associated with lung and other cancers. Lung cancer cells that exhibit high ALDH1 expression displayed the ability to proliferate and self-renew and were also chemotherapy resistant, all properties of TICs [31]. CD44 is extensively used as a TIC marker for prostate, pancreas and colorectal cancers [32, 33]. In a study done by Leung et al. non-small cell lung carcinomas expressing CD44 were positive for stemness markers Oct-4, Sox-2, and Nanog, but CD44- cells did not express these stemness markers, suggesting a putative role of CD44 in TICs [34]. Oct-4, Nanog, and Sox-2 are candidate markers for bronchial carcinoid tumors, as these are pluripotency transcription factors [34]. In a study where Sox-2 expression was downregulated in the D121 lung cancer cell line, the metastatic potential was significantly suppressed [35]. Double knockdown of Nanog and Oct-4 in lung adenocarcinoma cells lead to suppression of metastatic potential, epithelial-to-mesenchymal transition (EMT) and tumorigenic ability, suggesting a critical role of these stemness genes in TIC [36].

The presumptive TIC fraction in the bronchial carcinoid tumorigenic population has not been previously well examined in terms of the expression of such stemness markers. First, we were able to show a strong uptake of riboflavin revealed by strong autofluorescence that proved valuable for demonstrating concentration of stem cell fraction in spheroids. Next, we identified and characterized the bronchial carcinoid TIC population by the expression of stemness markers, ALDH1, CD44, Oct-4, Sox-2 and Nanog, appropriate markers based on previous studies on neuroendocrine tumors [37]. Using immunofluorescence and immunohistochemical labeling, and western blot analysis, we demonstrated that the expressions of stem cell markers were substantially up-regulated in H727 and H720 bronchial carcinoid 3rd gen SP cells. These stem cell markers are thus indicative of an increased fraction of TIC. These analyses further helped to validate our culture approach and the underlying switch to a stemness phenotype in spheroid grown cells.

Considering that enhanced chemoresistance has been demonstrated to be representative of an increase in the tumor stem cell fraction, we tested the dose-dependent response to cisplatin, reported as IC50 of 0.23 μg/ml, on the viability of H727 and H720 third-generation and parental cells [38, 39]. Spheroids were shown to be significantly more resistant to cisplatin (~ 2 fold) in comparison to parental cells.

In addition, through H&E staining, we observed increased vasculature and stroma around the H727 and H720 spheroid cells in xenografts but less in xenografts generated from parental cells. There has been increasing evidence suggesting that TIC stimulate angiogenesis and increase the stromal content, further confirming the larger TIC population in spheroid culture compared to parental cell culture [40].

TIC lack homeostatic regulation and largely contribute to the rapid proliferative rate of tumors [41]. Treatment modalities that can target TIC, in addition to reducing the bulk of the tumor, have demonstrated to be more effective in reducing relapse, metastasis and treatment resistance as opposed to chemotherapy alone. In identifying novel therapeutics that could target the TIC fraction in bronchial carcinoids we reasoned that TIC localize to hypoxic niches where hypoxia can up-regulate a large number of genes promoting growth, invasion and metastasis. Previous studies have demonstrated the role of carbonic anhydrase IX (CAIX), up-regulated by hypoxia, in driving “stemness” related genes and in TIC expansion [42]. CAIX is commonly overexpressed in cancer cells due to the hypoxic conditions that pervade growing tumors. CAIX has shown to be a mediator of tumor proliferation and metastasis [43]. This led us to consider the pan carbonic anhydrase inhibitor, AZ, as a viable therapeutic. From previous work [11], we chose the potent multi-targeting anti-tumor isothiocyanate, SFN, with low intrinsic toxicity, for the combination.

Essentially, here we show that, although SFN alone is an effective anti-tumor agent the AZ + SFN combination is most effective in reducing cell viability of H727 and H720 generated spheroid cells in-vitro, and inhibiting the growth of tumors, in-vivo. We also noted that a lower dose of AZ and SFN alone also exhibited a potent therapeutic effect, thus favoring reduction in any tissue toxicity that would be normally produced with a higher dose of monotherapy. Thus the results obtained here demonstrate the enhanced potency of the AZ + SFN combination for targeting the TIC fraction in bronchial carcinoids selected by serial spheroid passaging.

We then asked if the AZ + SFN combination could have clinical potential if we had a orthotopic model of bronchial carcinoids. This lung site is from where metastatic progression might ensue [44]. We successfully developed this model and confirmed the anti-tumor potency of the AZ + SFN combination. Histological analysis suggested major loss of tumor cells, phenotypic changes, and possible stromal replacement. Next we developed an appropriate PDX model for bronchial carcinoids. We were able to derive a PDX model by generation of spheroids and direct xenografting orthotopically in lung. To enable this orthotopic model using patient derived bronchial carcinoids we facilitated tumor take and growth, based on other studies in our lab, by co-culturing bronchial carcinoid patient spheroids with fetal lung fibroblasts to provide a supporting stroma. Since the patient tumor derived spheroids possess excellent tumorigenic potential and could grow orthotopically, this approach merits further development. Therefore from a bronchial carcinoid therapy perspective, our evidence that SFN, and enhancement by the AZ + SFN combination, significantly reduced the TIC tumorigenic potential in both AC and TC variants of bronchial carcinoids, portends clinical relevance.

To explain why the AZ + SFN combination would be most effective as a therapeutic regimen we offer the following. Recent studies support SFN as a potent anti-tumor agent against multiple signaling pathways in diverse cancers and targeting of mitochondrial functions where normal cells are protected [45–50]. Expression of carbonic anhydrase IX (CAIX) is highly relevant to rapidly growing and aggressive tumor cells adapting to the acidic microenvironment in rapidly growing tumors in order to maintain a more intracellular physiological pH, and thus inhibitors of CAIX can potently inhibit tumor cell viability [51, 52]. Here we show that AZ alone is an effective but more modest growth inhibitory agent. As regulation of pH is a complex process [52] more precise CAIX inhibitors may be required. Nevertheless, AZ is already in clinical use for other conditions and thus can be readily repurposed. In comparison, SFN, as a recognized epigenetic modulator that can suppress miRNA and growth factors supporting cancer stem cells in diverse cancers [53, 54], as well as targeting survival pathways, is significantly more potent as an anti-tumor agent. Recent studies on pancreatic cancer demonstrating that SFN induces ROS formation via metabolic effects may account for the observed anti-tumor effects similar to those reported here on bronchial carcinoids [55]. Moreover, importantly, we show that SFN alone significantly downregulates stemness in bronchial carcinoids, further enhanced by the AZ + SFN combination. Therefore, we posit that the combination would place added physiological stress on tumor cells and especially the ability of TIC to survive and maintain growth. Furthermore, in support of this notion, in xenograft studies reported here we show that the AZ + SFN combination more rapidly affected tumor growth from earliest stages after xenografting (see Fig. 6). Finally, considering that in our novel lung orthotopic model the anti-tumor effects were repeatedly demonstrated, exploiting the orthotopic model, that recapitulates the microenvironment of bronchial carcinoids, could serve to investigate SFN and the AZ + SFN combination on the malignant progression of bronchial carcinoids.

## Conclusions

In summary, in-vitro culture of bronchial carcinoid cell lines under suspension stem cell like culture conditions yielded spheroids that could be passaged with increased clonogenicity and by the 3rd generation showed significantly increased expression of stemness markers including uptake of riboflavin, a stem cell marker. Notably, the 3rd gen SP showed a significant increase in TIC. Applying this spheroid approach to patient bronchial carcinoids, and including a supporting mesenchyme, facilitated the development of a novel orthotopic xenograft model with an efficiency of 100%. The 3rd gen SP cells readily formed tumors in immunocompromised mice both subcutaneously and orthotopically in lung. Treatment with SFN was highly growth inhibitory in-vivo and could be potentiated by addition of AZ. Thus, the AZ + SFN combination proved most effective in inhibiting bronchial carcinoid tumor growth. Evidence was obtained that after treatment of the bronchial carcinoid derived xenografts by SFN and by further enhancement with AZ in combination there was a significant reduction in the TIC fraction and a marked reduction in the tumorigenic potential of bronchial carcinoids. As these agents are readily amenable for therapy they are therefore quite promising for further evaluations including clinical trials exploiting this novel therapeutic approach.

## Data Availability

The data analyzed during this study are included in this published article.

## Abbreviations

AC: Atypical carcinoid
ATCC: American type culture collection
AZ: Acetazolamide
CAIX: Carbonic anhydrase IX
CDDP: Cisplatin
D: Diameters
EMT: Epithelial-to-mesenchymal transition
FBS: Fetal bovine serum
hEGF: Epidermal growth factor
hFGF: human fibroblastic growth factor
MRI: Magnetic resonance imaging
NET: Neuroendocrine tumor
PA: Parental cells
PBS: Phosphate buffer saline
PBST: PBS-Tween
PDX: Patient-derived xenograft
SFN: Sulforaphane
SickKids: Hospital for Sick Children
SP: Spheroids
TC: Typical carcinoid
TIC: Tumor initiating cells
Trypsin-EDTA: Trypsin-(Ethylenediaminetetraacetic acid)
